# The GPI-Anchored GH76 Protein Dfg5 Affects Hyphal Morphology and Osmoregulation in the Mycoparasite *Trichoderma atroviride* and Is Interconnected With MAPK Signaling

**DOI:** 10.3389/fmicb.2021.601113

**Published:** 2021-02-10

**Authors:** Lea Atanasova, Dubraska Moreno-Ruiz, Clemens Grünwald-Gruber, Viktoria Hell, Susanne Zeilinger

**Affiliations:** ^1^Department of Microbiology, University of Innsbruck, Innsbruck, Austria; ^2^Department of Food Science and Technology, University of Natural Resources and Life Sciences, Vienna, Austria; ^3^Division of Biochemistry, University of Natural Resources and Life Sciences, Vienna, Austria; ^4^Core Facility Mass Spectrometry BOKU, University of Natural Resources and Life Sciences, Vienna, Austria

**Keywords:** *Trichoderma atroviride*, cell wall, mycoparasitism, mitogen-activated protein kinase, glycoside hydrolase family 76

## Abstract

The fungal cell wall is composed of a cross-linked matrix of chitin, glucans, mannans, galactomannans, and cell wall proteins with mannan chains. Cell wall mannans are directly attached to the cell wall core, while the majority of mannoproteins is produced with a glycosylphosphatidylinositol (GPI) anchor and then transferred to β-1,6-glucan in the cell wall. In this study, we functionally characterized the transmembrane protein Dfg5 of the glycoside hydrolase family 76 (GH76) in the fungal mycoparasite *Trichoderma atroviride*, whose ortholog has recently been proposed to cross-link glycoproteins into the cell wall of yeast and fungi. We show that the *T. atroviride* Dfg5 candidate is a GPI-anchored, transmembrane, 6-hairpin member of the GH76 Dfg5 subfamily that plays an important role in hyphal morphology in this mycoparasite. Alterations in the release of proteins associated with cell wall remodeling as well as a higher amount of non-covalently bonded cell surface proteins were detected in the mutants compared to the wild-type. Gene expression analysis suggests that transcript levels of genes involved in glucan synthesis, of proteases involved in mycoparasitism, and of the Tmk1 mitogen-activated protein kinase (MAPK)-encoding gene are influenced by Dfg5, whereas Tmk3 governs Dfg5 transcription. We show that Dfg5 controls important physiological properties of *T. atroviride*, such as osmotic stress resistance, hyphal morphology, and cell wall stability.

## Introduction

The use of antagonistic microbes for plant disease management is a sustainable alternative to chemical fungicides (Benítez et al., 2004). *Trichoderma* (teleomorph *Hypocrea*, Ascomycota) species are efficient necrotrophic mycoparasites. These fungi are able to parasitize and kill other fungi, a behavior that makes *Trichoderma* species attractive biocontrol agents for plant disease control (Mukherjee et al., 2013). Necrotrophic mycoparasitic fungi attack and lyse other fungal cells and feed on their dead cell contents (Deacon and Deacon, 2006; Druzhinina et al., 2011; Karlsson et al., 2017). The different stages during the mycoparasitic attack include sensing of the prey fungus, attachment to the prey’s hyphae, degradation of the prey’s cell wall, and ultimately killing of the prey (Chet et al., 1981; Inbar and Chet, 1992; Rocha-Ramirez et al., 2002; Lu et al., 2004). Several genes coding for chitinases, β-(1,4)-, β-(1,3)-, and β-(1,6)-glucanases, and proteases are induced under mycoparasitism-related growth conditions, and the respective enzymes degrade the cell wall of the prey fungus during mycoparasitism (Benítez et al., 2004; Kubicek et al., 2011; Gruber and Seidl-Seiboth, 2012; Atanasova et al., 2013).

The cell wall is a major component of fungal cells and plays a critical role in fungal biology. It protects the cell from the environment but at the same time allows the fungus to sense and get into contact with its environment. The fungal cell wall is composed of a matrix of cross-linked biopolymers such as chitin, glucans, mannans, galactomannans, and cell wall proteins, and its exact composition is species-dependent (see Free, 2013). Many proteins being integrated into the cell wall are synthesized as glycosylphosphatidylinositol (GPI)-anchored proteins and have enzymatic, antigenic, and adhesive functions. Only proteins passing through the secretory pathway (proteins with an N-terminal signal peptide) are subject to GPI anchoring (Free, 2013). The GPI anchor is a glycolipid structure that functions to anchor proteins into the outer plasma membrane leaflet [and to the luminal leaflet of the endoplasmic reticulum (ER) and Golgi apparatus while the protein is in transit to the plasma membrane] (Thomson et al., 2003). In *Neurospora crassa*, the N-linked galactomannan is required for the incorporation of integral cell wall proteins into the cell wall matrix (Maddi and Free, 2010; Maddi et al., 2012b). The majority of cell wall mannans and galactomannans are derived from posttranslational modifications of cell wall proteins, and the posttranslational glycosylation of cell wall proteins is essential for cell wall formation (Jin, 2012; Free, 2013).

In *Saccharomyces cerevisiae*, mating and filamentous growth (FG) are governed by the mitogen-activated protein kinases (MAPKs) Fus3 and Kss1, respectively (Madhani and Fink, 1997). Both, Fus3 and Kss1, control Ste12, a transcription factor that acts as a regulator in both mating and invasive growth. In the FG mode, *Candida albicans* and other pathogenic fungi synthesize a variety of cell surface proteins (Chaffin, 2008) to adjust their adherence characteristics and create cell surface variegation important for virulence (Halme et al., 2004; Nather and Munro, 2008). Most FG pathway proteins are also involved in other MAPK pathways in the cell (Birkaya et al., 2009) and regulate more than one cellular process (Martínez-Soto and Ruiz-Herrera, 2017). The Fusp/Kss1p MAPK cascade shows a high degree of conservation in filamentous fungi, which, in contrast to yeast, nearly always possess a single ortholog of Fus3/Kss1 only (Rispail and Di Pietro, 2010). Accordingly, three MAPKs are encoded in the genomes of *Trichoderma* species. These belong to the so-called pheromone response/FG, cell wall integrity (CWI), and osmoregulation pathways (Medina-Castellanos et al., 2014). The respective MAPKs are named Tmk1, Tmk2, and Tmk3 in *T. atroviride* with their corresponding yeast orthologs being Kss1/Fus3, Slt2, and Hog1 (Mendoza-Mendoza et al., 2003; Delgado-Jarana et al., 2006; Reithner et al., 2007; Zeilinger and Omann, 2007).

Glycoside hydrolases of the GH76 family are found within bacteria and fungi. Bacterial GH76 enzymes specifically recognize and cleave α-1,6-mannans. However, data on the catalytic mechanisms of fungal GH76 enzymes of this family as well as on their biochemical and structural properties are scarce. Vogt et al. (2020) recently reported that the GH76 subfamily Dfg5, which is crucial for the maturation of fungal GPI-anchored cell wall proteins, is the largest of all GH76 subfamilies and ubiquitous in ascomycetes. It has been shown that Dfg5 enzymes catalyze the transfer of GPI-anchored proteins from the plasma membrane to the glycan meshwork as a key step in cell wall biogenesis of filamentous fungi (Maddi et al., 2012b; Muszkieta et al., 2019; Vogt et al., 2020). Two homologous genes encoding GPI-anchored membrane GH76 proteins, *DCW1* and *DFG5*, were shown in *S. cerevisiae* to be crucial for cell growth and cell wall biogenesis (Kitagaki et al., 2002). In *C. albicans*, it was demonstrated that the Dfg5p protein is N-terminally mannosylated and is needed for the hypha-specific gene *HWP1* being expressed under alkaline conditions (Spreghini et al., 2003). Because Dfg5p is a cell surface protein, the authors suggested a role in generating or transmitting an external signal that governs the expression of hypha-specific genes. In *N. crassa*, two of nine GH76 proteins were suggested to be involved in cell wall biosynthesis by cleaving and transferring N-linked outer-chain mannans onto the cell wall (Maddi and Free, 2010; Maddi et al., 2012b). Deletion of six of its seven GH76 paralogs in the human pathogen *Aspergillus fumigatus* was not lethal but abolished the transfer of GPI-anchored galactomannans to the cell wall (Muszkieta et al., 2019).

In this study, we show that the *T. atroviride* homolog of *N. crassa* Dfg5 is a member of the GH76 Dfg5 subfamily required for hyphal morphogenesis and osmoregulation in this prominent mycoparasite. The Δ*dfg5* mutants showed reduced radial growth with tight colonies, thin and hyper-branched hyphae, and patchy deposition of chitin in their cell wall. Altered release of non-covalently entrapped cell surface proteins was detected in the mutants’ fermentation broth and after disruption of its cell walls, suggesting that secretion of cell wall proteins might be affected by the *T. atroviride* Dfg5 protein. Finally, we show that expression of *dfg5* and the MAPK-encoding *tmk1* and *tmk3* genes is co-dependent.

## Materials and Methods

### Strains and Conditions of Cultivation

*Trichoderma atroviride* strain P1 (ATCC 74058) was used as the wild-type (WT) throughout this study. All fungal mutants used, except Δ*tmk3*, which is derived from *T. atroviride* IMI 204060 (Esquivel-Naranjo et al., 2016), are derived from this strain. *Rhizoctonia solani* (teleomorph *Thanatephorus*, Basidiomycota) was employed as a fungal prey in confrontation assays. *Escherichia coli* strains JM109 and Stellar (Clontech, TaKaRa) were used for plasmid amplification and construction. Fungi were grown and maintained on potato dextrose agar (PDA; Sigma) at 25°C under cycling daylight unless otherwise stated. Here, 20-mm^2^ mycelial plugs of *T. atroviride* were inoculated on PDA supplemented with 1 M NaCl or 50 mM sorbitol for assessment of osmotic stress resistance. Cultures with at least three biological replicates per strain were grown at 25°C in darkness, and colony development was measured daily (colony diameter in mm). For gene expression analysis of MAPK genes (*tmk1, tmk2*, and *tmk3*), *dfg5*, chitin synthase genes (*chs1* and *2*), genes involved in glucan synthesis (*fks1*, *gel1*, and *smi1*), and *prb1* and *s8* protease genes (for protein IDs, see Supplementary Table 3), strains were cultivated in potato dextrose broth (PDB; Sigma) at 25°C and 150 rpm. For analysis of Dfg5 subfamily gene expression, WT and Δ*dfg5* deletion mutant 8-1 were cultivated in PDB with and without addition of 5 μg/ml Congo red (CR). Cultures were sampled after 5 days. To test the effect of Dfg5 on cell wall stability, strains were cultivated on PDA containing either 278.7 μg/ml CR or 5 μg/ml caspofungin (CAS). Biomass of the WT and Δ*dfg5* mutants was assessed in PDB with and without addition of 10 μg/ml CAS under the same conditions as described above.

### Growth Rate, Hyphal Morphology, and Dual Confrontation Assays

For growth rate evaluation, *T. atroviride* WT and mutants were cultured at the above described conditions at 25°C in darkness and their radial growth was measured every 24 h for 7 days. For the analysis of hyphal cell wall, a 10-mm^2^ culture plug from a 7-day-old PDA plate was placed on a new PDA plate. The strains were incubated in darkness and, after 5 days, plates were examined under a Leica inverted LSM SP5 laser scanning confocal microscope. Small pieces of fungal colony edges were cut out from the plate and inverted over a drop of 0.5% calcofluor white (CFW). Double image scanning was done using UV and bright field mode. Images obtained in bright field were used for hyphal diameter analysis, measuring 100 hyphae of each strain with Fiji software (Schindelin et al., 2012). For hyphal branching, 1-μl drops of a spore solution with a concentration of 1.2 conidia/μl were inoculated on PDA plates and incubated at 25°C in 12-h cycling light/dark conditions for 24 h. One hundred conidia were examined under a Nikon SMZ1500 stereomicroscope, and pictures were taken using a Nikon eclipse Ts2 inverted microscope, and a Leica inverted LSM SP5 laser scanning confocal microscope.

For confrontation assays, *T. atroviride* WT and mutants were co-cultivated with *R. solani* or self-confronted (control) on PDA plates covered with a cellophane membrane. Here, 20-mm^2^ culture plugs were placed on the opposite side of the agar plate 1 cm from the edge of the plate. Cultures were incubated at 25°C for 7 days under cyclic daylight (16 h indirect sunlight, 8 h darkness). The inhibition of *R. solani* growth was calculated by assuming that the distance between the inoculation plugs of both fungi is 100%. The growth of *Trichoderma* in the self-confrontation control was set as a zero inhibition rate. Growth inhibition of *R. solani* was calculated as the percentage of *Trichoderma* growth corrected for the growth against itself.

### Construction of Plasmids and Transformation of *T. atroviride*

*Trichoderma atroviride dfg5* gene deletion strains were generated by applying the split marker technique. The *E. coli hph* gene (mediating hygromycin B resistance) under control of the *Aspergillus nidulans gpdA* promoter and *trpC* terminator was used as the selection marker (Gruber and Zeilinger, 2014). Double joint-PCR was employed for PCR amplification and fusion reactions (Yu et al., 2004) using primers listed in Supplementary Table 1. Here, ∼1,500 bp upstream and downstream of the *dfg5* coding region (Ta130206) were amplified using genomic DNA of *T. atroviride* and primers promoter dfg5fw/dfg5Rv (upstream) and terminator dfg5fw/dfg5Rv (downstream) (Supplementary Table 2). *hph* split marker fragments were PCR amplified from pBluescript II KS (-)_hph plasmid (Mach et al., 1994). DNA fragments (3 μg) were used for the transformation of fungal protoplasts and emerging transformants selected in the presence of 200 μg/ml hygromycin B. After purification to mitotic stability by three rounds of single spore isolation, deletion of the *dfg5* gene was confirmed by PCR genotyping using the primer pair P5Fw/P5Rv, located before the upstream flanking region, outside of the integrated deletion cassette, and in the middle of the resistance marker, and P6Fw/P6rv amplifying the *dfg5* gene (Supplementary Table 2). For the generation of *dfg5* overexpressing mutants, the *dfg5* open reading frame was amplified from genomic DNA and fused with NEBuilder^®^ HiFi DNA Assembly Kit (New England Biolabs, Germany) downstream of the constitutively active *Trichoderma reesei pki1* promoter (Schindler et al., 1993) replacing the *gpr1*-mEGFP gene fusion in the P*pki1*:*gpr1*-mEGFP*:Tgpr1* construct (Atanasova et al., 2018). The primers used for the assembly are given in Supplementary Table 2. The P*pki1*:*dfg5* construct was linearized by *Bgl*II and introduced into *T. atroviride* WT and Δ*dfg5* background. Transformants emerging on 300 μg/ml nourseothricin sulfate (Jena Bioscience, Germany) were purified by three rounds of single spore isolation and the integration of the respective expression cassettes in their genomes confirmed by genotyping PCR.

### Analysis of Gene Expression by RT-qPCR

Here, 5 μg of RNA was treated with DNase I and reverse transcribed using the RevertAid H Minus First Strand cDNA Synthesis Kit (Thermo Fisher Scientific, Waltham, USA) according to the manufacturer’s protocol with a combination of oligo(dT) and random hexamer primers. Three biological replicates were pooled prior to RNA extraction. PCR reactions were performed in triplicate on a Bio-Rad (Hercules, CA) iCycler IQ using IQ SYBR Green Supermix (Bio-Rad, Hercules, CA, United States), standard MgCl_2_ concentration (3 mM), and a final primer concentration of 100 nM in a total volume of 25 μl. Primer sequences with the corresponding parameters are provided in Supplementary Table 3. The PCR protocol comprised an initial denaturation step (2 min at 95°C) followed by 40 cycles of denaturation (5 s at 95°C), annealing (20 s, for Tm, see Supplementary Table 3) and extension (65°C for 10 s). qPCR efficiency was determined using triplicate reactions from a dilution series (1, 0.1, 10^–2^, and 10^–3^) of cDNA. The given slopes in the IQ5 Optical system Software v2.0 (Bio-Rad, Hercules, CA, United States) were used to calculate the amplification efficiency. Expression ratios and standard errors were determined using Pfaffl test model in REST (Pfaffl et al., 2002) with *sar1* as reference gene (Brunner et al., 2008).

### Phylogenetic Analysis

We examined the position of Dfg5 family proteins of *Trichoderma* species in the phylogenetic tree according to their sequence similarity to *N. crassa* OR74A, *A. nidulans* FGSC A4, *S. cerevisiae* S288C, *C. albicans* SC5314, and *T. atroviride* IMI206040. Protein sequences were obtained from the genomes available on the JGI portal^1^, AspGD^2^, NCBI^3^ ^,4^. The MUSCLE analysis tool was applied for the alignment of the predicted amino acid sequences (Edgar, 2004). Phylogenetic interference was performed using recombination network analysis in SplitsTree4 program (Huson and Bryant, 2006) implementing Equal Angle method. Bayesian phylogram of 100 Dfg5 orthologs being most similar to *T. atroviride* Ta130206 obtained from the NCBI database (accessed on May 15, 2020) was performed using the Dayhoff amino acid substitution model. Metropolis-Coupled Markov Chain Monte Carlo (MCMCMC) sampling was done with MrBayes v3.2.5 (Ronquist et al., 2012) applying two simultaneous runs of four incrementally heated chains that generated 10 million generations in total. Two completely independent analyses starting from different random trees were performed. After dropping the first 25% of the trees (burn-in), trees were summarized. Bayesian posterior probabilities (PPs) were obtained from the 50% majority-rule consensus of trees sampled every 100 generations after burn-in. PP values < 0.95 were not considered significant, and values <0.9 were not displayed in the resulting cladogram.

### Confocal Laser Scanning Microscopy

Hyphal morphology and branching of *T. atroviride* WT and mutants were analyzed using a Leica inverted LSM SP5 laser scanning confocal microscope. Here, 0.5% CFW solution was used for fungal cell wall visualization. CFW was imaged by excitation with UV laser line, and fluorescence was detected in a range of 380–480 nm. Images were analyzed using Fiji software (Schindelin et al., 2012). Package IBM SPSS Statistics 24 was used to perform one-way analysis of variation (ANOVA), and least significance difference (LSD) was set at *p* = 0.05.

### Total Cell Wall Protein and Non-covalently Bonded Cell Surface Protein Content

Cell walls were isolated after cultivation of strains in PDB for 96 h at cycling daylight, 150 rpm, and 25°C. The mycelia were thoroughly washed twice with sterile water, and 100 mg of the fresh biomass was shock-frozen in liquid nitrogen and ground to a fine powder. The total cell walls were washed extensively as described by Moreno-Ruiz et al. (2009) to remove non-covalently linked proteins. Further, cell walls were resuspended in 100 μl 1 M NaOH and incubated at 100°C for 10 min, and the suspension was then neutralized by addition of 100 ml 1 M HCl and centrifuged. The protein concentration of the non-covalently bonded proteins and cell wall supernatants were determined with the Bradford protein assay using bovine serum albumin (BSA) as a reference.

### Liquid Chromatography-Electrospray Ionization-Mass Spectrometry

Liquid chromatography-electrospray ionization-mass spectrometry (LC-ESI-MS) was performed to identify the secreted proteins and/or proteins that were released or were not integrated into the fungal cell wall during cultivation in PDB for 96 h at cycling daylight, 150 rpm, and 25°C. Proteins secreted/released in an amount of culture broth equivalent to one containing 5 mg of dry fungal biomass were precipitated with acetone and dissolved in 15 μl of phosphate-buffered saline (PBS). The samples mixed with the same amount of 2× Laemmli buffer were then loaded on a stain-free sodium dodecyl sulfate-polyacrylamide gel electrophoresis (SDS-PAGE) gel (Biorad, Germany). Gel zones at the same range, which showed a different band pattern between the WT and Δ*dfg5*-8-1, were excised from the gel (Supplementary Figure 1) and digested with trypsin (duplicates were pooled). Five blocks were analyzed separately resulting in 10 independent samples (A–E for WT and Δ*dfg5*-8-1, respectively; see Supplementary Figure 1). Proteins were in gel S-alkylated with iodoacetamide and digested with trypsin (Promega, Mannheim, Germany). Peptides were extracted and directly loaded on a Thermo Acclaim PepMap300 RSLC C18 separation column (2-μm particle size, 150 mm × 0.075 mm) using a Thermo Acclaim PepMap μ-precolumn with 0.1% formic acid as the aqueous solvent. After applying a gradient from 6% B [B: 80% acetonitrile (ACCN)] to 40% B in 30 min, a 10-min gradient from 40% B to 90% B at a flow rate of 0.3 μl/min was performed that facilitates elution of large peptides. A QTOF MS (Bruker maXis 4G ETD) equipped with the captive spray source in positive ion, DDA mode (=switching to MSMS mode for eluting peaks) was used for detection. The six highest peaks were selected for fragmentation from recorded MS scans (range: 150–2,200 Da). The instrument was calibrated using ESI calibration mixture (Agilent, Santa Clara, CA, United States). The analysis files were converted (using Data Analysis, Bruker) to XML files, which are suitable for performing an MS/MS ion search with ProteinScape (Bruker, MASCOT embedded). The files were searched against the *T. atroviride* database (downloaded from Uniprot 6. Nov 2017), and the search results for the individual gel zones were combined (Supplementary Tables 4, 5). The hits were then manually curated. GPI anchors and transmembrane (TM) domains were predicted using PredGPI online tool (Pierleoni et al., 2008) and TMHMM Server v. 2.0 (Krogh et al., 2001), respectively.

## Results

### *Trichoderma atroviride* Dfg5 Is a Transmembrane Glycosylphosphatidylinositol-Anchored Six-Hairpin Member of the GH76 Dfg5 Subfamily

**FIGURE 1:**
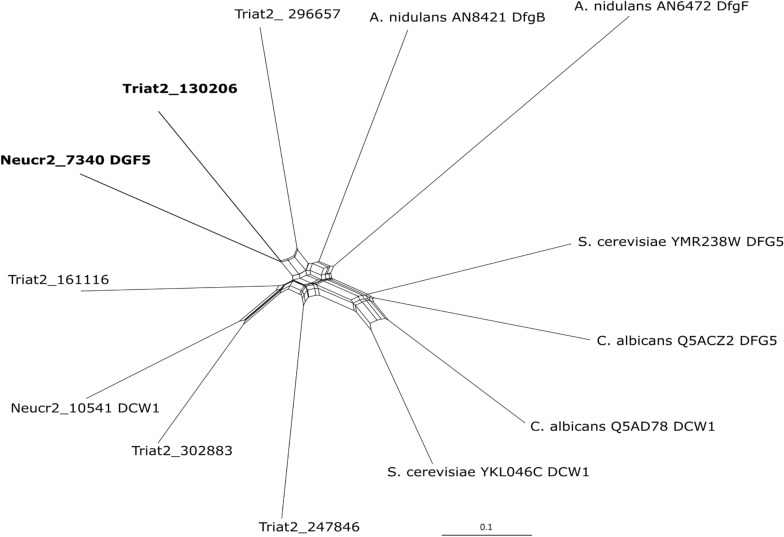
Phylogenetic inference of Dfg5 orthologs from *N. crassa* OR74A (Neucr2), *A. nidulans* FGSC A4, *S. cerevisiae* S288C, *C. albicans* SC5314, and *T. atroviride* IMI206040 using recombination network analysis in SplitsTree4 (Huson and Bryant, 2006) program. The tool was used as a framework for interfering with the phylogenetic relation between the multiple *T. atroviride* Dfg5 orthologs and already characterized Dfg5 and Dcw1 proteins.

**FIGURE 2:**
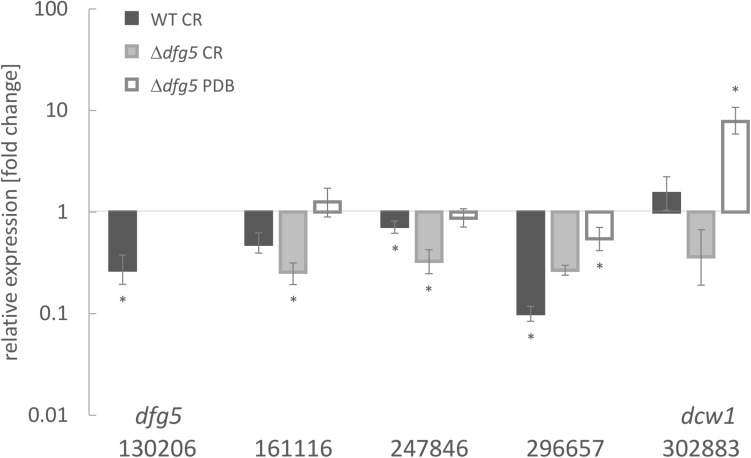
Transcript levels of Dfg5 subfamily member genes in *T. atroviride* wild-type (WT) and a Δ*dfg5* deletion mutant upon cultivation in potato dextrose broth (PDB) with and without the addition of the cell wall stressor Congo red (CR). The asterisks (*) indicate statistical significance in regulation of the respective genes (–1.5 < log2(FC) > 1.5, where FC indicates fold change) toward the untreated control and the *sar1* normalizer. The lines above the bars indicate standard error (SE) as calculated by the Relative Expression Software Tool REST using SE estimation *via* a Taylor algorithm (Pfaffl et al., 2002).

In a genome-wide comparative transcriptome analyses of the *T. atroviride* Δ*tmk1* mutant (Reithner et al., 2007) and the WT, we detected reduced transcript levels of a gene encoding a GPI-anchored member of glycoside hydrolase family 76 (GH76; EC 3.2.1.101; Ta130206^5^) (Atanasova and Zeilinger, unpublished). The gene contains three exons and two introns; in the deduced protein sequence, one TM domain was predicted. The protein further bears a six-hairpin glycoside transferase domain, with an alpha/alpha toroid fold and six alpha-hairpins arranged in a closed circular array (IPR008928). GH76 family members are enzymes found in bacteria and fungi. Recently, Vogt et al. (2020) reported a crystal structure of a fungal GH76 protein from the Dfg5 subfamily in complex with a reassembled GPI core glycan. Their sequence analyses showed that the Dfg5 subfamily is an isofunctional subfamily of GH76 proteins, which clearly differs from other bacterial, fungal, and archaeal GH76 subfamilies with annotated α-1,6-mannanase function (Vogt et al., 2020). Furthermore, Muszkieta et al. (2019) argued that recombinant *Sc*Dcw1 protein, a homolog of Dfg5, showed no transglycosylase or hydrolase activity, thus it likely does not represent an α-mannanase enzyme as known for bacterial GH76. BLAST searches within the NCBI database and across the genomes of the species with characterized Dfg5/Dcw1 family members were performed. Phylogenetic recombination network analysis using SplitsTree4 (Huson and Bryant, 2006) with some of the published orthologs (Figure 1) confirmed that Ta130206 is the closest ortholog of *N. crassa* Dfg5. Expression analysis of all five Dfg5 orthologs in a *T. atroviride* Δ*dfg5* deletion mutant (generation and further properties, see below) revealed a compensation effect of the *dcw1* ortholog (Ta302883) under neutral growth conditions (PDB). During cell wall stress (growth in the presence of CR), however, neither *dfg5* nor its orthologs were significantly upregulated in the WT, but also not in the mutant (Figure 2). Dfg5 orthologs have already been functionally characterized in several fungal organisms such as *S. cerevisiae* (Kitagaki et al., 2002), *C. albicans* (Spreghini et al., 2003), *N. crassa* (Maddi et al., 2012b), *A. nidulans* (de Groot et al., 2009), and *A. fumigatus* (Muszkieta et al., 2019). In yeasts, Dfg5 is required for cell wall biogenesis during bud formation and for FG (Kitagaki et al., 2002), whereas *N. crassa* Dfg5 was proposed to cross-link N-linked oligosaccharide-associated galactomannan and the cell wall glucan/chitin matrix, which results in an effective covalent cross-linking of glycoproteins into the cell wall matrix (Maddi et al., 2012b). In *A. fumigatus*, single gene deletions revealed that *dfg3* plays the most important morphogenetic role in this gene family, whereas a sextuple-deletion mutant resulted in lack of galactomannan in the cell wall and severe growth defects (Muszkieta et al., 2019). We generated a predicted homology model of *T. atroviride* Dfg5 from features of the target-template alignment, where the *Chaetomium thermophilum* protein showed the highest template quality and was selected for model building (Supplementary Figure 2).

Comprehensive Bayesian analysis of 100 orthologous sequences being most similar to *T. atroviride* Dfg5 (Supplementary Figure 3) obtained from the NCBI database using BLAST as well as additional *N. crassa* Dfg5 and Dcw1 proteins revealed a separation of Dfg5/Dcw1 family members into two Hypocreales clades following a clear grouping based on the nutritional modes of the respective fungi. The proteins derived from *Trichoderma* spp. all possess a GPI anchor and share a common ancestor with those from entomopathogenic Hypocreales members. Respective proteins from Nectriaceae fell into two groups, one forming a well-supported clade with GPI anchor and TM domain and the second one containing paralogs without GPI anchor or TM domain being related to some *Colletotrichum* orthologs. A third large clade combined the rest of *Colletotrichum* spp. or paralogs (Glomerellales), and species from other Sordariomycetes. *N. crassa* Dfg5 and Dcw1 emerged as an out-group.

### Dfg5 Is Required for Proper Hyphal Morphogenesis of *T*. *atroviride*

To functionally characterize Dfg5 in *T. atroviride*, *dfg5* gene deletion mutants were generated. Transformation of fungal protoplasts with the deletion cassette resulted in 16 hygromycin B-resistant transformants. Of those, two transformants, Δ*dfg5*-2-2 and Δ*dfg5*-8-1, showed homologous integration of the deletion construct and hence complete deletion of the *dfg5* open reading frame. For the generation of complemented strains and strains overexpressing *dfg5*, a construct harboring *dfg5* fused to the strong constitutive *pki1* promoter (P*pki1*:*dfg5* construct) was transformed into both Δ*dfg5* mutants and the *T. atroviride* WT strain, respectively. However, despite several attempts, reintegration of *dfg5* into the Δ*dfg5* mutant was unsuccessful, while transformation of the WT with the overexpression construct yielded one mitotically stable mutant, named *dfg5*OE. We speculate that the failure to produce complementation mutants was due to the altered cell wall of the Δ*dfg5* mutants (see below).

**FIGURE 3:**
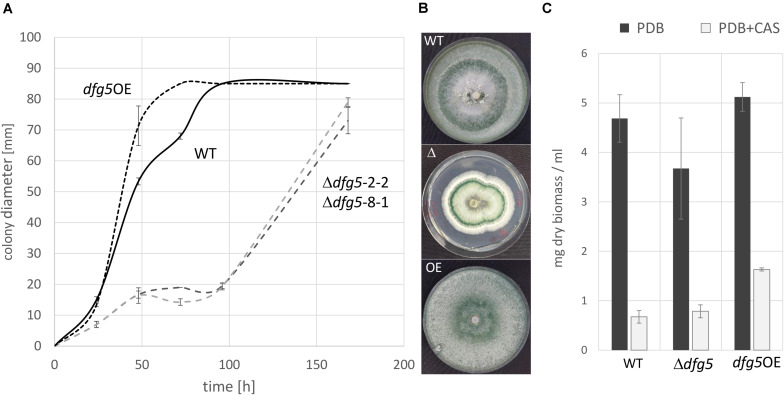
Growth rate **(A)** and macromorphology **(B)** of *T. atroviride* Δ*dfg5* deletion mutants and the wild-type (WT) upon cultivation on potato dextrose agar (PDA). **(C)** Biomass production of Δ*dfg5*, the overexpression strain *dfg5*OE, and the WT upon cultivation in liquid culture [potato dextrose broth (PDB) or PDB supplemented with the glucan synthase inhibitor caspofungin (CAS)]. Due to the highly similar phenotype of the two Δ*dfg5* mutants (Δ*dfg5*-2-2 and Δ*dfg5*-8-1), results of Δ*dfg5*-8-1 are presented in the following unless otherwise stated.

**FIGURE 4:**
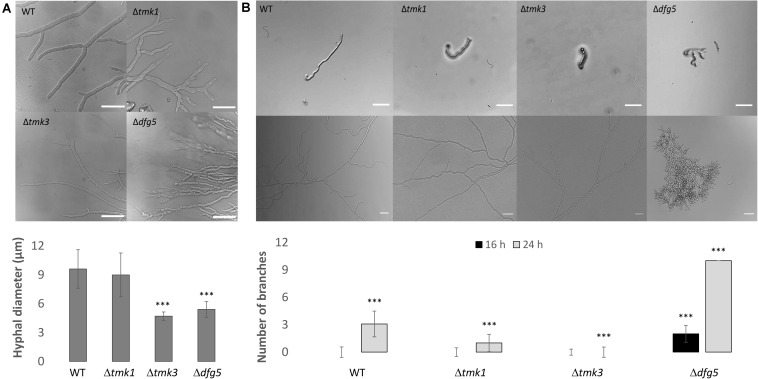
**(A)** Hyphal morphology of *T. atroviride* Δ*dfg5*, Δ*tmk1*, and Δ*tmk3* deletion mutants as well as of the wild-type (WT) after growth on potato dextrose agar (PDA) for 48 h. Δ*dfg5* mutants showed thin hyphae with a “curled” hyphal phenotype. Images were taken using a laser scanning confocal microscope. The bars annotate 50 μm. The bar chart illustrates hyphal diameters of the tested strains, which were measured using Fiji software. *p-*value (^∗∗∗^) ≤ 0.001. **(B)** Hyphal branching in WT as well as Δ*tmk1*, Δ*tmk3*, and Δ*dfg5* mutants. Bright field microscopy was used to assess branching in colonies that developed from one single conidium. One conidium of each strain was inoculated on PDA and observed after 16 h (upper image row) and 36 h (lower image row) of incubation at 25°C. Scale bar: 10 μm. The bar chart illustrates the branching frequency of the tested strains after 16 and 24 h of incubation on PDA (the quantification at 36-h time point was unsuitable due to Δ*dfg5* mutant’s dense growth). *p-*value (^∗∗∗^) ≤ 0.001.

**FIGURE 5:**
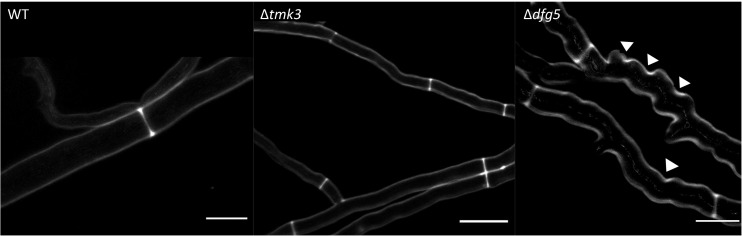
Confocal laser scanning fluorescence microscopy of *T. atroviride* wild-type (WT), Δ*tmk3*, and Δ*dfg5* mutants after staining their cell wall with 0.5% CFW solution. The Δ*dfg5* mutant hyphae showed a “curly” shape, which was associated with patchy cell wall accumulations. The arrows indicate sites with a thicker deposition of chitin in the cell wall. Scale bar: 50 μm.

Phenotypic analyses revealed that the two Δ*dfg5* mutants behaved highly similar, while they significantly differed from the WT in macro- and micro-morphology. Upon cultivation on solid complex medium (PDA) under daylight illumination, the deletion mutants formed small compact colonies with significantly less aerial hyphae than the WT and the *dfg5*-overexpressing strain (Figures 3A,B). In *T. atroviride*, conidia production was described to be light-triggered. Constant light results in continuous conidiation across the fungal colony, whereas cultivation under light–dark conditions leads to the formation of concentric rings of conidia (Steyaert et al., 2010). Interestingly, the formation of concentric conidial rings was more evident in the Δ*dfg5* mutants, while expression of *dfg5* under the strong constitutive *pki1* promoter only had a minor influence on the conidiation pattern (Figure 3B). Δ*dfg5* deletion mutants showed significantly reduced growth rates compared to the WT upon cultivation on PDA, whereas the overexpression mutant *dfg5*OE had higher hyphal elongation rates (Figure 3A). Biomass production in liquid culture (PDB) however was similar between Δ*dfg5*, *dfg5*OE, and the WT (Figure 3C). Microscopic examination of the hyphal phenotype at the colony periphery revealed that Δ*dfg5* mutants produced abnormally condensed mycelia with thinner but “curled” hyphae (Figure 4A). A detailed analysis of the branching behavior further revealed that *dfg5* deletion led to hyper-branched hyphae (Figure 4B). To visualize putative changes in cell wall structure of Δ*dfg5* mutants, we used the CFW dye as a stain that binds to the chitinous part of the cell wall and emits fluorescence. Patchy cell wall accumulations were evident in the Δ*dfg5* mutant under the laser scanning confocal microscope, which were not visible in the WT (Figure 5), evidencing a function of Dfg5 in the distribution of cell wall components and, subsequently, in hyphal morphology. Uneven distribution of cell wall components was also supported by CR staining (Supplementary Figure 4). A similar chitin accumulation in patches at the lateral walls, together with reduced apical growth and isotropic tip swelling, has recently been described for *T. atroviride* chitin synthase mutants Δ*chs5* and Δ*chs7* (Kappel et al., 2020).

### Δ*dfg5* Mutants Are Altered in Osmotic Stress Resistance and Mycoparasitic Activity

**FIGURE 6:**
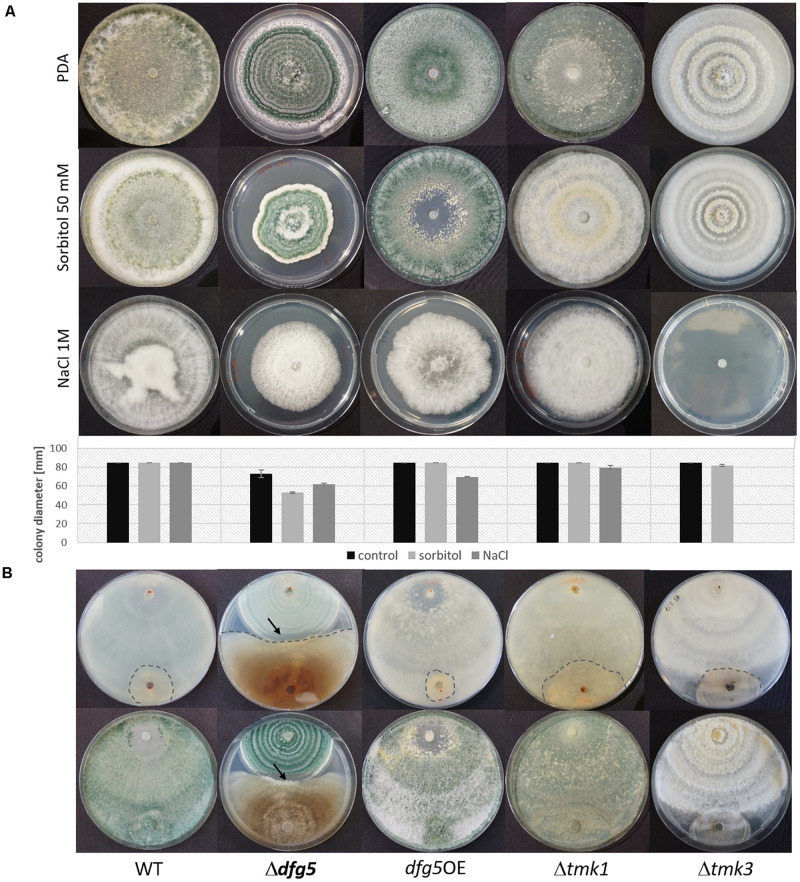
Involvement of *Dfg5* in the response to osmotic stress and in antagonism of *T. atroviride* against *R. solani*. **(A)** Cultures of Δ*dfg5*, *dfg5*OE, Δ*tmk1*, and Δ*tmk3*, as well as the wild-type (WT) on potato dextrose agar (PDA) without any stressor and on PDA supplemented with 50 mM sorbitol or 1 M NaCl. Plates were incubated at cycling daylight for 10 days at 25°C. Graphs represent the colony diameters of the tested strains under the different growth conditions (black bar, growth on PDA; light gray bar, growth in the presence of 50 mM sorbitol; dark gray bar, growth in the presence of 1 M NaCl) as an indicator of their stress sensitivity. Error bars represent standard deviations derived from at least three biological replicates. **(B)** Plate confrontation assays against *R. solani* (back and front sides of the plates are shown). The arrows mark the clearing zone devoid of sporulation of the Δ*dfg5* mutant upon contact with *R. solani*. Due to the highly similar behavior of the two Δ*dfg5* mutants, only results of Δ*dfg5*-8-1 are presented.

For *T. reesei* and *N. crassa*, it was previously reported that high osmolarity conditions affect the cell wall by strongly limiting glucan and chitin synthesis (da-Silva et al., 1994; Górka-Nieć et al., 2010). High osmolarity stress is sensed by the high-osmolarity glycerol (HOG) MAPK pathway that is activated by increased concentrations of different chemicals such as sorbitol and NaCl (Gustin et al., 1998). To test whether *dfg5* deletion affects the sensitivity of *T. atroviride* against sorbitol- and sodium chloride-mediated stress, the Δ*dfg5* mutants were cultivated with and without one of these stress inducers, and their behavior was compared to the WT, the *dfg5*OE strain, and the available MAPK-deficient mutants Δ*tmk1* (Reithner et al., 2007) and Δ*tmk3* (Esquivel-Naranjo et al., 2016). The presence of 50 mM sorbitol or 1 M NaCl resulted in impaired growth of Δ*dfg5*, whereas the WT and the Δ*tmk1* mutant were highly resistant against these two stressors (Figure 6A). The Δ*tmk3* and *dfg5*OE strains were barely affected by sorbitol but significantly affected by NaCl on which the Δ*tmk3* mutant was unable to grow (Figure 6A). The *dfg5*OE strain showed a growth reduction of ∼25% in the presence of 1 M NaCl. Interestingly, none of the strains tested (including the WT) was able to conidiate upon supplementation with 1 M NaCl, although cultivation was performed under cyclic daylight conditions.

To test the effect of Dfg5 on the cell wall stability of *T. atroviride*, strains were grown on PDA supplemented with 278.7 μg/ml CR or 5 μg/ml CAS. CR dye complexes with (helical) chitin chain parts, thereby resulting in a loss of cell wall rigidity, which is due to an impaired lateral interaction between the helices (Kopecká and Gabriel, 1992). CAS, however, is an echinocandin that acts as an inhibitor of the β-1,3-glucan synthase in fungal cell wall biosynthesis in a non-competitive manner. Inhibition of β-1,3-glucan synthesis leads to growth reduction, an increase in osmotic sensitivity, and may even result in cell lysis (Valiante et al., 2015). Addition of CAS resulted in highly impaired growth of all strains tested (Supplementary Figure 5). In contrast, CR reduced the growth of the WT, *dfg5*OE, and Δ*tmk1* stains by 20, 13, and 27%, respectively, while growth of Δ*dfg5* and Δ*tmk3* mutants was not affected (Supplementary Figure 5A). For Δ*dfg5* mutants, this might be explained by the insufficient intercalation of CR with the already disrupted Δ*dfg5* cell wall due to uneven chitin distribution (Figure 5 and Supplementary Figure 4). The role of Dfg5 in *T. atroviride* cell wall stability was further investigated in liquid cultures. After 96 h of cultivation with CAS, both Δ*dfg5* mutants did not show any statistically significant changes in biomass production compared to the WT, while the *dfg5*OE strain showed enhanced growth (Figure 3C). However, the addition of CAS resulted in reduced transcript levels of the chitin synthase encoding gene c*hs1*, the glucan synthase encoding gene *fks1* and *smi1*/*knr4* (regulator of 1,3-β-glucan synthase activity and cell wall formation in *N. crassa* and yeasts; Enderlin and Selitrennikoff, 1994; Hong et al., 1994) in all tested strains, while mRNA levels of the chitin synthase *chs2* were increased under this condition. Transcription of the *gel1* glucanosyltranferase was negatively affected in the Δ*dfg5* mutants but not in the WT in the presence of CAS compared to the condition without the stressor (Supplementary Figure 5C).

To test whether *dfg5* impacts *T. atroviride* mycoparasitism, *R. solani* was used as fungal prey in plate confrontation assays. Similar to the WT, the *dfg5*OE mutant was able to attack and completely overgrow the prey within 7 days. In contrast, Δ*dfg5* mutants were less successful in overgrowing the prey fungus and showed a lower growth inhibition of *R. solani*, which indicates a reduced antagonistic potential (Figure 6B). Yet, due to the altered growth rate of the Δ*dfg5* mutants, the observed reduction in mycoparasitic overgrowth could also be a consequence of the mutants’ growth defect. Nevertheless, Δ*dfg5* mutants formed a clearing zone when confronted with *R*. *solani*, where they did not conidiate upon the contact with the prey fungus (Figure 6B and Supplementary Figure 6).

### Δ*dfg5* Mutants Release Enhanced Amounts of Non-covalently Bonded Cell Surface Proteins

To investigate whether *T. atroviride* Dfg5 affects protein integration into the cell wall, cell walls of the WT and Δ*dfg5* mutants cultivated in PDB for 5 days were analyzed for the total protein content in the cell walls and for the amount of non-covalently bonded cell wall proteins. Protein incorporation into the cell wall was not affected by *dfg5* deletion; however, the mutants showed an elevated release of the non-covalently bonded proteins after cell surface disruption (Supplementary Figure 7). The total amount of proteins secreted into the culture broth was lower in the mutants compared to the WT (Supplementary Figure 1). Finally, proteins released into the culture medium by the WT and Δ*dfg5* were qualitatively analyzed by LC-ESI-MS. From a total of 76 secreted proteins detected by this analysis, 29 proteins were secreted only by Δ*dfg5*, whereas seven were secreted only by the WT strain. Analysis of proteins exclusively secreted by Δ*dfg5* (Table 1) revealed that 35% of them are GPI-anchored proteins, among which we detected two members of GH17 endoglucanases, three GH16 cell wall glucanosyltransferases, a GH72 glucanosyltransferase, a GH28 endopolygalacturonase, gamma-glutamyltransferase, a protein with a CBM9 domain, and two uncharacterized GPI-anchored proteins. A highly secreted GPI-anchored GH92 α-1,2-mannosidase, a putative peptidase S8, and a GPI-anchored cell wall organization protein were detected with a much higher number of tryptic peptides identified in the Δ*dfg5* mutant than in the WT culture supernatant (Supplementary Tables 4, 5). These results suggest an altered secretion of non-covalently bonded proteins by the Δ*dfg5* mutant.

**TABLE 1:** Proteins secreted specifically by the *T. atroviride* wild-type (WT) or the Δ*dfg5* mutant after 5 days of cultivation in potato dextrose broth (PDB).

**Protein identification**	**JGI protein ID**	**MASCOT score**		**Peptides**		**SC (%)**		**TM**	**GPI**
		**Δ*dfg5***	**WT**	**Δ*dfg5***	**WT**	**Δ*dfg5***	**WT**		
Carboxylesterase	285336	641	0	26	0	34.5	0	0	0
**GH16; GPI-glucanosyltransferase**	**154960**	**419.2**	**0**	**29**	**0**	**28.2**	**0**	**0**	**1**
PL7_4; putative alginate lyase	315643	307.3	0	12	0	47.8	0	0	0
GH28; endopolygalacturonase	28947	267.6	0	13	0	22.2	0	0	0
Phosphoglycerate mutase	155981	264.1	0	29	0	21.8	0	0	0
**Uncharacterized protein**	**218916**	**263.6**	**0**	**14**	**0**	**18.6**	**0**	**0**	**1**
**GH17; putative GPI-anchored cell wall β-1,3-endoglucanase**	**298716**	**260.7**	**0**	**18**	**0**	**14.1**	**0**	**0**	**1**
GH75; endo-chitosanase	216890	247	0	9	0	19	0	0	0
Ribonuclease	301022	229.5	0	14	0	27.3	0	0	0
**Gamma-glutamyltransferase**	**234843**	**206.2**	**0**	**9**	**0**	**15.7**	**0**	**0**	**1**
Uncharacterized protein	320017	171.3	0	12	0	12.8	0	0	0
Glycerophosphoryl diester phosphodiesterase	42544	166.7	0	4	0	13.2	0	0	0
Putative serine protease S28	296922	150.5	0	10	0	11.3	0	0	0
Uncharacterized protein	212071	141.4	0	10	0	10.3	0	0	0
GH71, α-1,3-glucanase	300906	132.1	0	3	0	9.1	0	0	0
AA1_3; multicopper oxidase; laccase	40409	104.8	0	5	0	5.1	0	0	0
**Uncharacterized protein**	**224396**	**104.5**	**0**	**4**	**0**	**9.9**	**0**	**1**	**1**
GH75; endo-chitosanase	54365	98.6	0	4	0	18.4	0	0	0
GH12; putative endoglucanase	44429	89.2	0	4	0	19.3	0	0	0
**β-1,3-glucanosyltransglycosylase; GH72**	**297466**	**88.9**	**0**	**4**	**0**	**7.7**	**0**	**0**	**1**
Related to epoxide hydrolase N terminus	45073	85.3	0	3	0	5.4	0	0	0
GH114; endo-α-1,4-polygalactosaminidase	300014	79.8	0	3	0	9.3	0	1	0
Protein with FAD binding domain	132671	79.7	0	2	0	4.3	0	0	0
**CBM18-GH16; GPI cell wall glucanosyltransferase**	**299218**	**79.3**	**0**	**5**	**0**	**4.2**	**0**	**0**	**1**
**GH16; GPI-cell wall glucanosyltransferase**	**311401**	**69.4**	**0**	**2**	**0**	**5.3**	**0**	**1**	**1**
Vacuolar protease A VPA1	137844	60.4	0	2	0	10.6	0	0	0
**GH17; putative GPI-anchored cell wall b-1,3-endoglucanase**	**301307**	**43.4**	**0**	**2**	**0**	**7.3**	**0**	**0**	**1**
**Protein with CBM9 domain**	**296843**	**36.7**	**0**	**5**	**0**	**16.7**	**0**	**0**	**1**
Carboxylesterase	137796	30.5	0	3	0	5.5	0	0	0
GH88; putative unsaturated glucuronyl hydrolase	46148	0	240	0	10	0	25.2	0	0
GH71, α-1,3-glucanase	302588	0	141.5	0	6	0	10.4	0	0
Peptidase M, neutral zinc metallopeptidases	299629	0	105.7	0	3	0	8.2	0	0
GH15-CBM20; glucoamylase	213708	0	92.2	0	3	0	4.6	0	0
Predicted small secreted cysteine-rich protein	282317	0	79.4	0	3	0	8.8	0	0
GH71-CBM24-CBM24; α-1,3-glucanase	44123	0	68.2	0	4	0	3.8	0	0
Putative aspartic protease	33651	0	61.2	0	3	0	11.1	0	0

*The MASCOT score, number of peptides identified for the specific protein, sequence coverage (SC), number of transmembrane (TM) domains, and lipid anchor glycosylphosphatidylinositol (GPI) are shown. GPI-anchored proteins are shown in bold.*

### Deletion of *dfg5* Impacts Mitogen-Activated Protein Kinase, Glucan Synthase, and Mycoparasitism-Relevant Protease Gene Transcription

**FIGURE 7:**
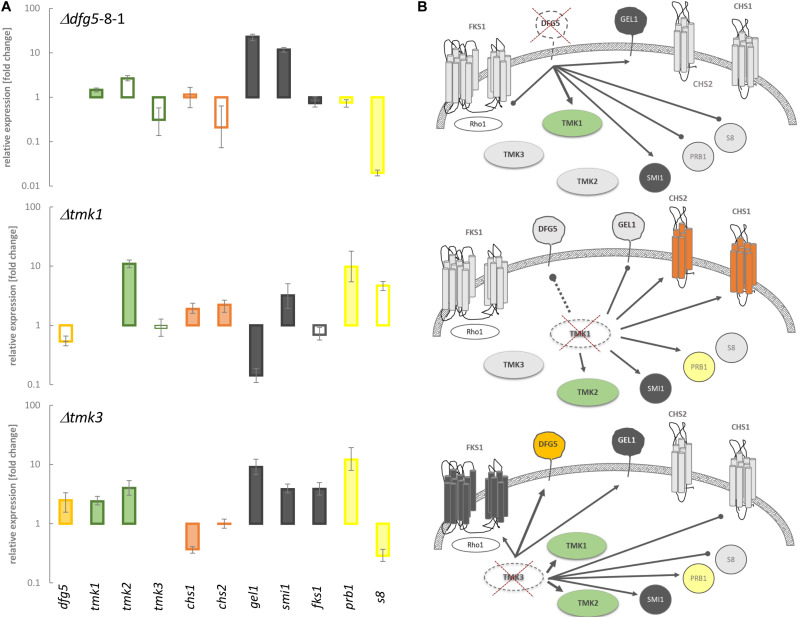
Transcript levels of genes putatively involved in signaling and cell wall remodeling in *T. atroviride*, as well as of two proteases known to be involved in mycoparasitism, in Δ*dfg5* and the Δ*tmk1* and Δ*tmk3* mitogen-activated protein kinase (MAPK)-deficient mutants, normalized to the transcriptional levels of the wild-type (WT). **(A)** Transcriptional regulation of *tmk1*, *tmk2*, and *tmk3* MAPK-encoding genes, *dfg5*, two chitin synthase genes (*chs1* and *2*), genes involved in glucan synthesis (*fks1*, *gel1*, and *smi1*), and *prb1* and *s8* protease genes in the indicated mutants normalized to the WT. The filled bars indicate statistically significant regulation of the respective genes [–1.5 < log2(FC) > 1.5, where FC indicates fold change] toward the untreated control and the normalizer; unfilled bars represent non-significant changes. The lines above the bars indicate standard error (SE) as calculated by the Relative Expression Software Tool REST using SE estimation *via* a Taylor algorithm (Pfaffl et al., 2002). **(B)** Schematic representation of the proposed interplay between MAPKs and the genes involved in cell wall remodeling upon deletion of *dfg5*, *tmk1*, and *tmk3*, respectively. The connecting lines between the objects annotate statistically significant activating (arrows) or suppressing (dots) relations between particular proteins in a hyphal cell. The single connecting dashed line symbolizes non-significant regulation of *dfg5* in the Δ*tmk1* mutant. The colored (green, orange, red, and dark gray) and light gray forms annotate transcriptional upregulation and downregulation of a particular gene in a certain strain, respectively. Crossed dashed-line objects indicate deletion of this particular gene.

The MAPK Tmk3 has previously been reported to contribute to CWI maintenance in *T. atroviride* and *T. reesei*, and for the latter species, a similar role has been identified for Tmk2 as well (Wang et al., 2014; Esquivel-Naranjo et al., 2016). To get insights into a putative interplay between Dfg5 and MAPK signaling, we performed a transcriptional analysis of the three *T. atroviride* MAPK-encoding genes (*tmk1*, *tmk2*, and *tmk3*), the *dfg5* gene, two chitin synthase-encoding genes (*chs1* and *chs2*), glucan synthase *fks1*, 1,3-β-glucanosyltranferase *gel1*, the regulator of 1,3-β-glucan synthase and putative interaction partner of yeasts Slt2 (Basmaji et al., 2006) (homolog of Tmk2 in *T. atroviride*) *smi1*/*knr4*, and the mycoparasitism-relevant *prb1* and *s8* protease-encoding genes in the WT, the Δ*dfg5* mutants, and the available Δ*tmk1* (Reithner et al., 2007) and Δ*tmk3* (Esquivel-Naranjo et al., 2016) MAPK-deficient mutants. *Tmk1* as well as *gel1* and *smi1* gene transcription was upregulated in the Δ*dfg5* mutant compared to the WT, while the glucan synthase *fks1* and both protease-encoding genes showed reduced transcription compared to the WT (Figure 7A). Enhanced transcript levels of *prb1*, as well as *smi1*, *chs1*, and *chs2*, were also observed in the Δ*tmk1* mutant. However, *dfg5* gene transcription was enhanced in the Δ*tmk3* mutant, similar to genes involved in glucan synthesis and *tmk1* and *tmk2* gene expression (Figure 7B).

Based on the obtained transcription data, we propose that Dfg5 suppresses the expression of *tmk1* in the WT. Concurrently, Tmk1 might positively influence the expression of *dfg5* and *gel1*, whereas it suppresses chitin synthase and protease gene transcription. Moreover, deletion of *dfg5* resulted in downregulation of both *prb1* and *s8* protease genes and a (not significant) reduction of *tmk3* transcript levels (Figure 7A), while Tmk3 negatively impacts *dfg5* mRNA levels. The stimulating effect on *tmk1* transcription that further governs the expression of chitin synthases and genes involved in glucan synthesis in both Δ*dfg5* and Δ*tmk3* mutants might explain the similar physiological changes observed in both strains in regard to osmotic and salt stress tolerance (Figure 6).

## Discussion

The fungal cell wall is fundamental for the growth, survival, and morphogenesis of fungal organisms, providing a protective barrier against environmental conditions such as heat, cold, desiccation, and osmotic stress, but also in protection against microbial attack (Free, 2013). For pathogenic fungi, the cell wall is also crucial for virulence and pathogenicity as it mediates adhesion, thereby being essential for host tissue invasion and insulation against host defense. In the mycoparasite *T. atroviride*, coiling and adhesion to host hyphae are among the processes involved in the mycoparasitic attack (Lu et al., 2004; Druzhinina et al., 2011). In this study, we identified a GPI-anchored GH76 candidate (Ta130206) as a member of the Dfg5 subfamily that impacts hyphal morphogenesis and cell wall properties of this mycoparasite. A homologous protein model revealed 14 beta-helixes, of which one is both GPI anchor and TM domain. Phylogenetic recombination network analysis of the *T. atroviride* and other characterized Dfg5 orthologs confirmed that Ta130206 is a direct *N. crassa* Dfg5 ortholog. Deletion of Ta130206 (*dfg5*) resulted in a significant upregulation of the *dcw1* ortholog in *T. atroviride*. However, under cell wall stress conditions, regulation of all five *dfg* family orthologs was suppressed and no compensation effect was detected in the Δ*dfg5* mutant. Further phylogenetic analysis revealed that Dfg5 orthologs from other *Trichoderma* species all possess a GPI anchor and share a common ancestor with those from entomopathogenic Hypocreales, whereas respective proteins from Nectriaceae form a well-supported clade with GPI anchor and TM domain and a clade of paralogs without GPI anchor or TM domain.

*T. atroviride* Δ*dfg5* mutants formed compact colonies with condensed mycelia and thin, hyper-branched hyphae with altered cell wall characteristics. Their growth rates were significantly reduced compared to the WT, while the overexpression mutant *dfg5*OE showed faster hyphal extension rates. Δ*och*-1 deletion mutants, which lack the α-1,6-mannosyltransferase OCH-1 that is needed in *N. crassa* for the synthesis of galactomannans attached to N-linked oligosaccharides of cell wall proteins, also exhibited a tight colonial phenotype (Maddi and Free, 2010). Δ*och-1* deletion mutants were hypothesized to exhibit a hyphal elongation disorder, which is similar to the phenotype of a Δ*dcw1*, Δ*dgf5* double mutant of *N. crassa* (Maddi et al., 2012b). Growth reduction and morphological changes including thinner hyphae have also been described for *T. atroviride* chitin synthase-deficient mutants such as Δ*chs1* and Δ*chs2* (Kappel et al., 2020). Furthermore, the patchy chitin accumulation in the cell wall of *T. atroviride* Δ*dfg5* mutants is similar to chitin synthase mutants Δ*chs5* and Δ*chs7* (Kappel et al., 2020). In *in vitro* antagonistic assays, *T. atroviride* WT and the *dfg5*OE mutant were able to attack and completely overgrow the phytopathogen *R. solani*, whereas Δ*dfg5* mutants had reduced antagonistic activity. Taken together, these data suggest that, similar to chitin synthases, Dfg5 is involved in dynamic cell wall remodeling and might contribute to a successful mycoparasitic attack.

Fungal cell walls consist of chitin and chitosan, glucans, mannans, and/or galactomannans, as well as glycoproteins. Glucan biosynthesis occurs *via* plasma membrane-associated glucan synthases, which eject their products through the plasma membrane into the cell wall space. Similarly, chitin is synthesized by plasma membrane-associated chitin synthases and also extruded into the extracellular cell wall space as a linear polymer (Free, 2013). In *N. crassa*, the cell wall proteins are tied into the matrix by glycosylhydrolases/glycosyltransferases that cross-link cell wall polymers together (Free, 2013). Mutants lacking α-1,6-mannosyltransferase OCH-1, the enzyme that adds the initial mannose derived from galactomannan to the N-linked oligosaccharide, were shown to have defects in covalent incorporation of cell wall proteins but instead release GPI-anchored and non-GPI-anchored cell wall proteins into the culture supernatant (Maddi and Free, 2010). The *och-1* mutant was dramatically affected in growth and morphology, had a weakened cell wall, and was susceptible to lysis (Maddi and Free, 2010). Later on, it was found that *N. crassa* mutants that are lacking DFG5 and its ortholog DCW1 phenotypically resemble the OCH-1 mutant, both in morphology and in their inability for incorporation of cell wall proteins into the cell wall matrix (Maddi et al., 2012a,b). In the human pathogen *A. fumigatus*, a sextuple-deletion mutant devoid of all expressed *DFG* genes was missing galactomannan in the cell wall and had severe growth defects, and *DFG3* was shown to play the most important morphogenetic role in this gene family (Muszkieta et al., 2019). Dfg5 membrane proteins are required for the biosynthesis of the cell wall during bud formation in yeast and were shown to have overlapping specificities in *N. crassa*, *A. fumigatus*, *S. cerevisiae*, and *C. albicans* (Kitagaki et al., 2002, 2004; Spreghini et al., 2003; Maddi et al., 2012a,b; Muszkieta et al., 2019). Complementation of the Δ*dfg5/dcw1 S. cerevisiae* mutant by *A. fumigatus AfDFG3* resulted in a restored normal growth at 37°C, which was lost in the double yeast mutant, thereby showing that *AfDFG3* and *S. cerevisiae DCW1* genes share similar biological activities (Muszkieta et al., 2019). Changes in *T. atroviride* cell wall characteristics due to a lack of Dfg5 were evidenced by patchily distributed helical chain parts of chitin networks in the cell walls of Δ*dfg5* mutants that could be stained by CFW and CR. However, Δ*dfg5* mutants responded to the cell wall stressors CAS and CR in a similar way as the WT. Interestingly, none of the mutants with single deletions in *N. crassa* showed susceptibility to the cell wall perturbation reagents such as salt, detergent, or CAS (Maddi et al., 2012b).

With their alterations in osmotic stress resistance, in their colony phenotype, and with their thin hyphae, *T. atroviride* Δ*dfg5* mutants showed similarity to *T. atroviride* Δ*tmk3* mutants missing the Hog1-like MAP kinase Tmk3. Mutants lacking Tmk3 exhibited an enhanced sensitivity to osmotic and oxidative stresses, UV light, cell wall damaging agents, high temperature, and cadmium (Esquivel-Naranjo et al., 2016). High osmolarity stress is generally sensed by the HOG MAPK cascade that is triggered by elevated concentrations of different chemicals such as sorbitol and NaCl (Gustin et al., 1998). *T. atroviride* Δ*dfg5* mutants showed reduced growth in response to salt- and sorbitol-mediated stress. For *T. reesei* and *N. crassa*, it was indeed previously reported that high osmolarity conditions affect the cell wall by strongly limiting glucan and chitin synthesis (da-Silva et al., 1994; Górka-Nieć et al., 2010). Neither β-(1,3)-glucan nor alkali-insoluble β-(1,6)-glucan was found in *T. reesei* cultivated under high sorbitol conditions, and the chitin amount was more than twofold lower than upon cultivation in the absence of the stressor (Górka-Nieć et al., 2010). These data imply that the cell wall cannot be properly synthesized in the presence of high osmolarity, and let us speculate that the observed high sensitivity of mutants lacking Dfg5 to osmotic stress is due to alterations in their cell wall. In addition, the reduced *tmk3* transcript levels observed in Δ*dfg5* mutants could affect the HOG pathway and thereby also result in enhanced osmosensitivity. The HOG pathway including its central component, the Hog1/Tmk3 MAPK, is conserved across the fungal kingdom (Martínez-Soto and Ruiz-Herrera, 2017). Hog1 governs the adaption to a variety of environmental cues including oxidative, high osmolarity, ultraviolet radiation, and heat stress in yeast (Hohmann, 2002; Krantz et al., 2009). Besides governing the responses to osmolarity, fungicides, and oxidative stress, the Hog1 homolog OS-containing MAPK cascade acts as the output pathway of the circadian clock in *N. crassa* (reviewed in de Paula et al., 2008). Accordingly, *T. atroviride*, similar to *T. reesei* and *A. nidulans*, employs the Hog1/Tmk3 MAPK pathway for light signaling (Schuster et al., 2007; Esquivel-Naranjo et al., 2016; Yu et al., 2016). *T. atroviride* Tmk3 is immediately phosphorylated upon light exposure, which is dependent on the blue light receptor Blr1 (Esquivel-Naranjo et al., 2016). The extensive formation of multiple concentric rings by Δ*dfg5* and Δ*tmk3* deletion mutants upon exposure to cyclic light implies a putative connection between Tmk3 signaling and Dfg5. Nevertheless, the similar behavior in regard to osmotic and salt stress tolerance as well as their similar morphological features such as thin hyphae with the formation of condensed mycelium in the form of tight concentric rings that were observed in both Δ*dfg5* and Δ*tmk3* might as well be, at least partially, a result of the transcriptional interconnection with the Tmk1 pathway that also impacts the expression of chitin and glucan synthases in *T. atroviride*. This involvement of multiple MAPK signaling pathways in osmoregulation and CWI was also found in *C. albicans* (Brown et al., 2014). In this yeast, HOG signaling interacts with the CWI pathway, which contributes to the regulation of chitin and glucan synthesis, and the FG pathway, which regulates the expression of genes encoding protein-O-mannosyltransferases. Together, these MAPK cascades govern growth, morphogenesis, CWI, stress response, and virulence (reviewed by Brown et al., 2014).

Homologs of the *S. cerevisiae* MAPK FUS3 have been assigned to pheromone response and pathogenicity in filamentous fungi (Xu and Hamer, 1996). *T. atroviride* Δ*tmk1* mutants have reduced mycoparasitic activity but elevated chitinase enzyme activities and showed enhanced transcription of the chitinase-encoding genes *nag1* and *ech42* (Reithner et al., 2007). In this study, we further found that Tmk1 governs the transcription of genes involved in glucan synthesis and chitin synthase encoding genes except the β–1,3-glucan synthase gene *fks1. Fks1* transcription, similar to the other glucan synthases tested, turned out to be affected by *dfg5* gene deletion. In *S. cerevisiae*, disruption of the *fks1* gene reduced the amount of glucan in the cell wall, increased its chitin content, and activated the expression of the GPI-dependent cell wall mannoprotein CWP1 (Terashima et al., 2000). In the general cell wall damage repair in *S. cerevisiae*, the redistribution of FKS1 is dependent on the actin cytoskeleton and is mediated by the RHO1 GTPase switch and PKC1-activated MAPK cascade (Delley and Hall, 1999) that controls a highly conserved CWI signaling pathway. It is thus possible that in *T. atroviride*, Fks1 is affected by actin cytoskeleton conformational changes in the cell wall as a consequence of cell wall alterations caused by the absence of Dfg5.

In the model proposed for *N. crassa* that described the incorporation of glycoproteins into the cell wall *via* the α-1,6-mannan core, Dfg5 is supposed to bind on glycoproteins the N-linked galactomannan and integrate it into the cell wall matrix of glucan/chitin by cross-linking (Maddi et al., 2012b). *dfg5* gene deletion in *T. atroviride* led to an increased amount of non-covalently bonded cell surface proteins and in altered secretion of cell wall-associated proteins, from which 35% were GPI-anchored proteins that were not detected in the WT. Among others, GPI-glucanosyltransferases, GPI-endoglucanases, endo-chitosanases, and chitinases, a GPI-α-1,2-mannosidase, and a putative GPI-anchored cell wall organization protein are likely involved in cell wall remodeling and organization.

## Data Availability Statement

The original contributions presented in the study are included in the article/Supplementary Material, further inquiries can be directed to the corresponding author/s.

## Author Contributions

LA, DM-R, CG-G, and VH conducted the experiments. LA and SZ designed the study, analyzed the data, and wrote the initial manuscript. LA and DM-R prepared the figures and the Supplementary Material. All authors read, revised, and approved the manuscript.

## Conflict of Interest

The authors declare that the research was conducted in the absence of any commercial or financial relationships that could be construed as a potential conflict of interest.

## Footnotes

1 http://genome.jgi.doe.gov

2 http://www.aspergillusgenome.org/

3 https://www.ncbi.nlm.nih.gov/genome/

4 http://www.yeastgenome.org/

5 https://genome.jgi.doe.gov/Triat2/Triat2.home.html
