# Supplementary material for: The GPI-Anchored GH76 Protein Dfg5 Affects Hyphal Morphology and Osmoregulation in the Mycoparasite Trichoderma atroviride and Is Interconnected With MAPK Signaling
Source: Front Microbiol. 2021 Feb 10;12:601113. doi: 10.3389/fmicb.2021.601113 (PMC7902864; doi:10.3389/fmicb.2021.601113)
Supplement: Supplementary file 1 [file Data_Sheet_1.pdf]

Supplementary Material

1. Supplementary Figures and Table legends

1.1 Supplementary Figures

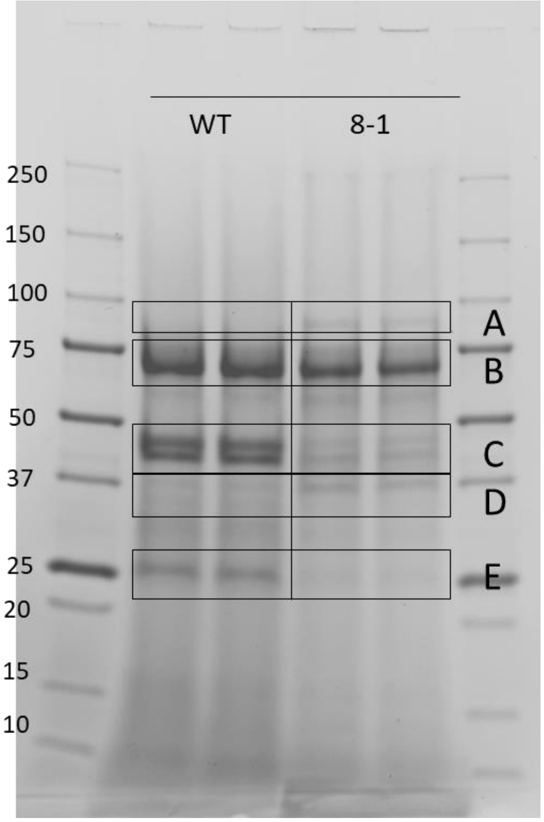

Supplementary figure 1: Sodium dodecyl sulfate–polyacrylamide gel (SDS-PAGE) electrophoresis from the WT and Δ*dfg5*-8-1 culture broths cultivated in PDB for 96h at cycling light, 150 rpm and 25°C. Proteins secreted from 5 mg of dry biomass were precipitated with acetone and dissolved in 15µl of PBS. The samples mixed with the same amount of 2x Laemmli buffer were loaded on the stain-free SDS-PAGE gel. WT and Δ*dfg5* replicates were combined and each gel block was analyzed separately (A-E).

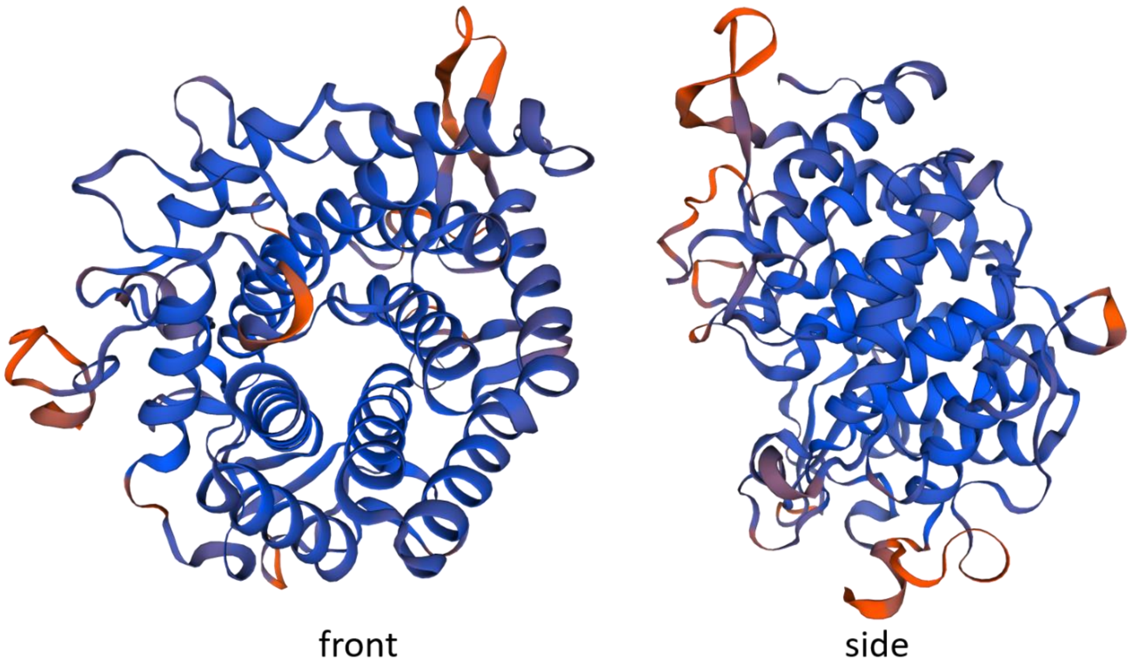

Supplementary figure 2: Structural protein model of *T. atroviride* Dfg5 protein using Swiss-model web tool for protein modeling, prediction and analysis (Waterhouse et al., 2018). The Swiss-model template library (SMTL version 2020-11-04, PDB release 2020-10-30) was searched for evolutionary related structures matching the target sequence. A homologous protein model of mannan endo-1,6-alpha-mannosidase was built based on the crystal structure of Dfg5 from *Chaetomium thermophilum* PDB 6ry0, GMQE (Global Model Quality Estimation) of 0.68, coverage of 0.88 and sequence identity of 44.44 %. The model is presented in colors based on the QMEAN model quality, that allows visualization of regions of the model that are well (blue) or poorly (red) modeled.

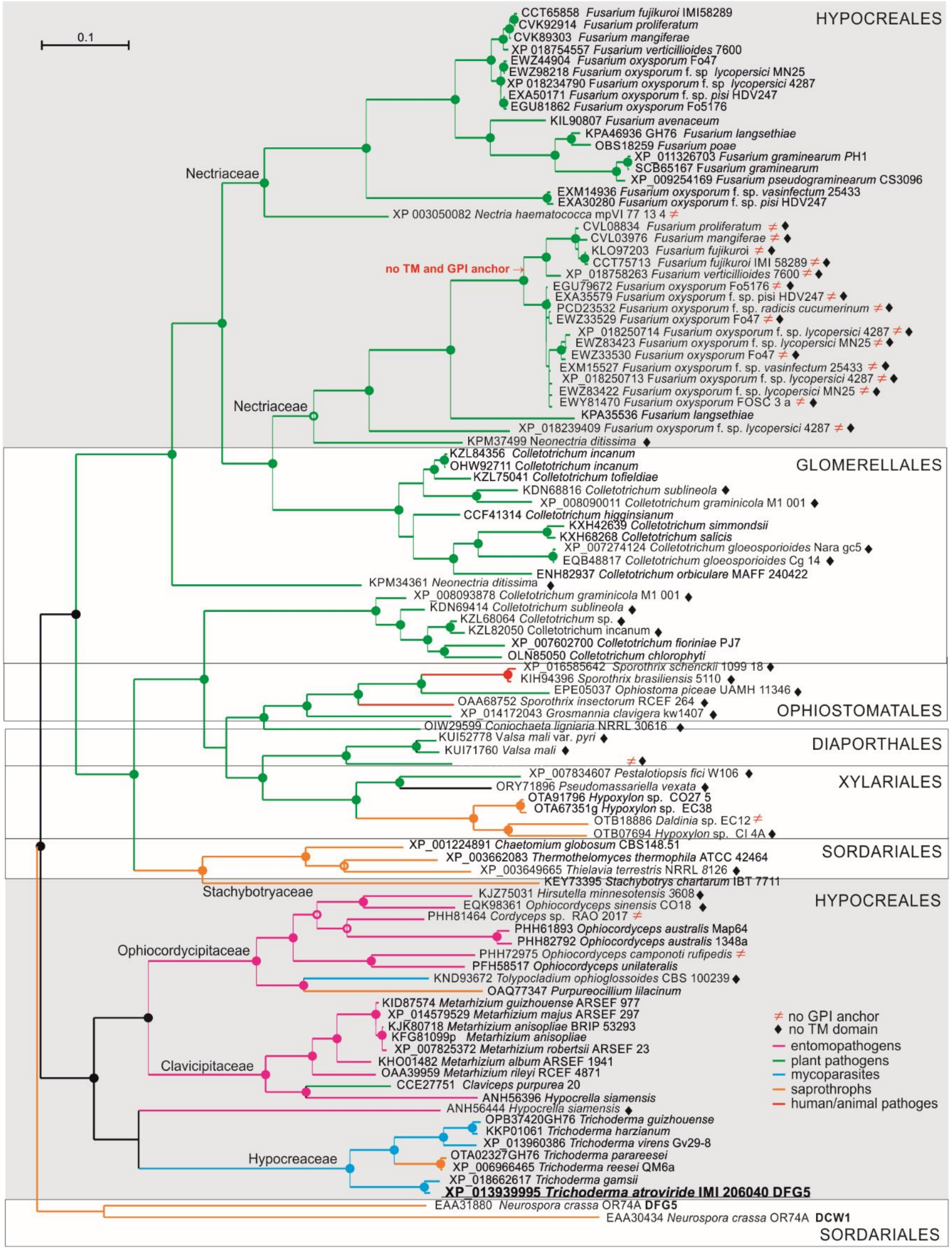

Supplementary figure 3: Bayesian phylogram of 100 Dfg5 orthologues being most similar to *T. atroviride* Ta130206 obtained from the NCBI database using BLAST. Analysis was run for ten million MCMC generations using Dayhoff amino acid substitution model. Posterior probability values lower than 0.95 were not considered significant. The phylogram reveals a separation of Hypocreales into two clades following a clear grouping based on the nutritional mode of the included fungi. *Trichoderma*

Dfg5 orthologs all possess a GPI-anchor and share a common ancestor with respective proteins from entomopathogenic Hypocreales. Full and empty nodes represent posterior probabilities over 0.94 and 0.90.

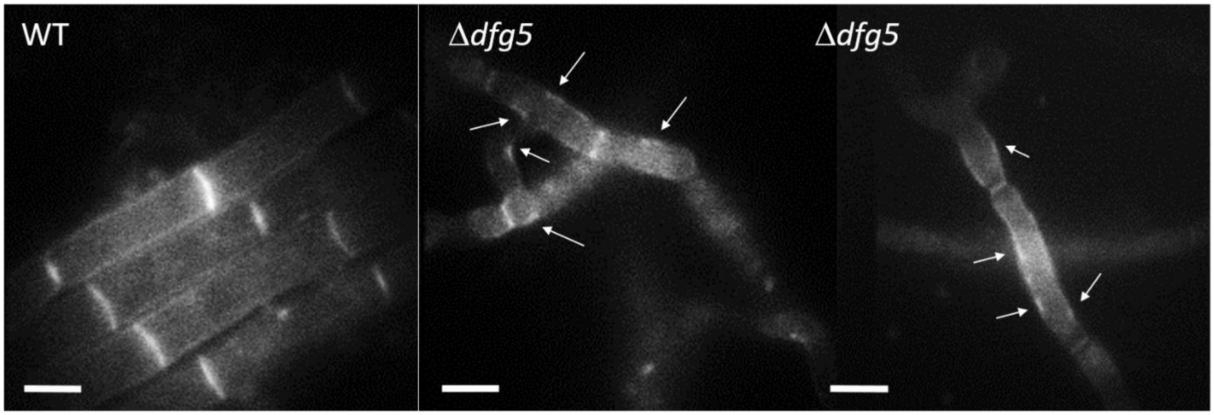

Supplementary figure 4: Confocal laser scanning microscopy of congo red stained hyphae of Δ*dfg5* and the wild type. Patchy cell wall accumulations (white arrows) were evident in Δ*dfg5* mutants, while the WT had a uniform, continuous cell wall. Fungal hyphae were visualized under a laser scanning confocal microscope upon 488 nm radiation after staining with 5µg/ml congo red solution. A plug of each culture was placed into 15 µ-slide 2-well glass bottom microscopy chambers coated with poly-lysine A and was cultivated in complete darkness for five days at 25 °C with addition of 100µl of PDB. The scale bar annotates 5µm.

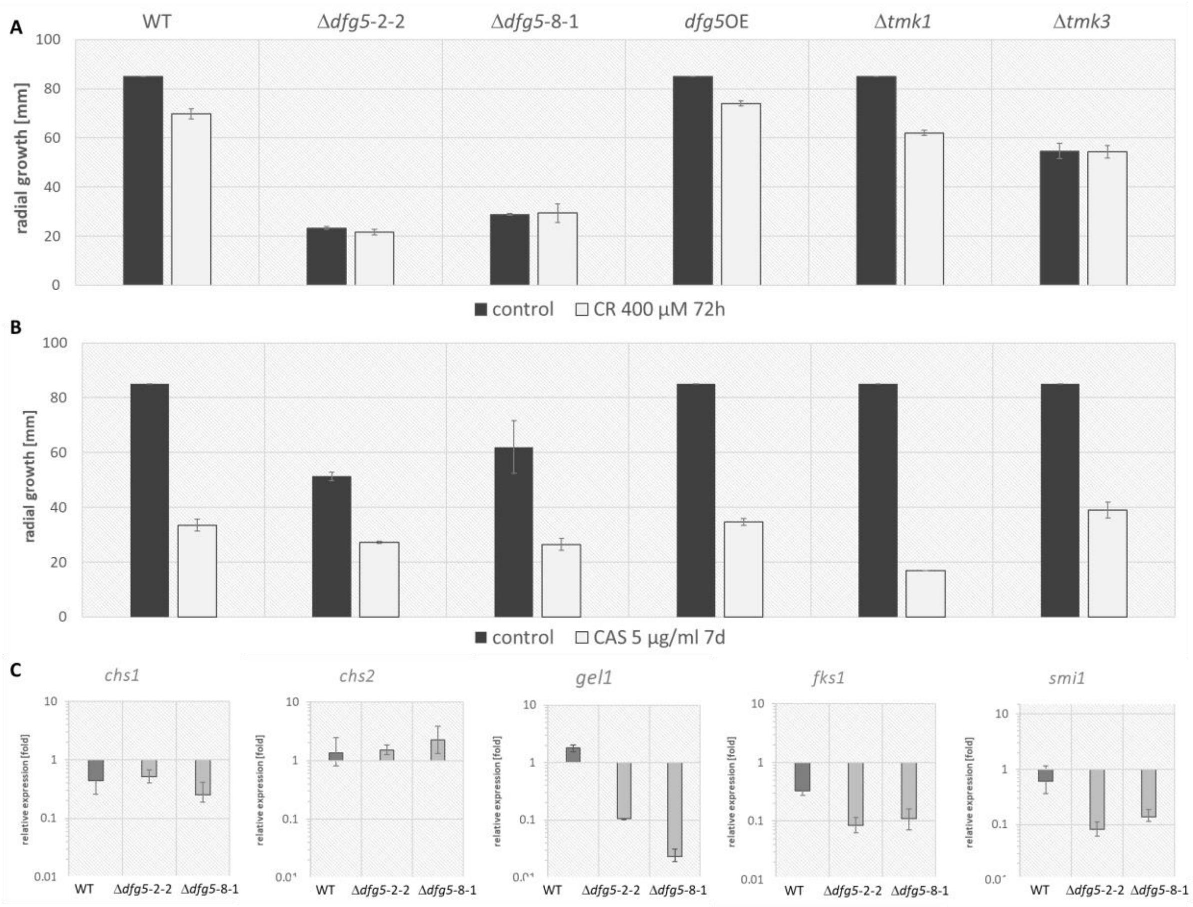

Supplementary figure 5: (A) Colony growth of Δ*dfg5*, *dfg5*OE, Δ*tmk1* and Δ*tmk3* mutants and the wild type on PDA supplemented with 278.7 µg/ml (400µM) congo red (CR) and (B) 5 µg/ml of the glucan synthase inhibitor caspofungin (CAS) after 72 hours of growth. (C) Transcript levels of selected chitin synthase-encoding genes and genes involved in glucan synthesis in the presence of CAS in wild type and Δ*dfg5* mutants normalized to the condition without stressor (PDB). The lines above the bars indicate standard error as calculated by the Relative Expression Software Tool REST using estimation via a Taylor algorithm (Pfaffl et al., 2002).

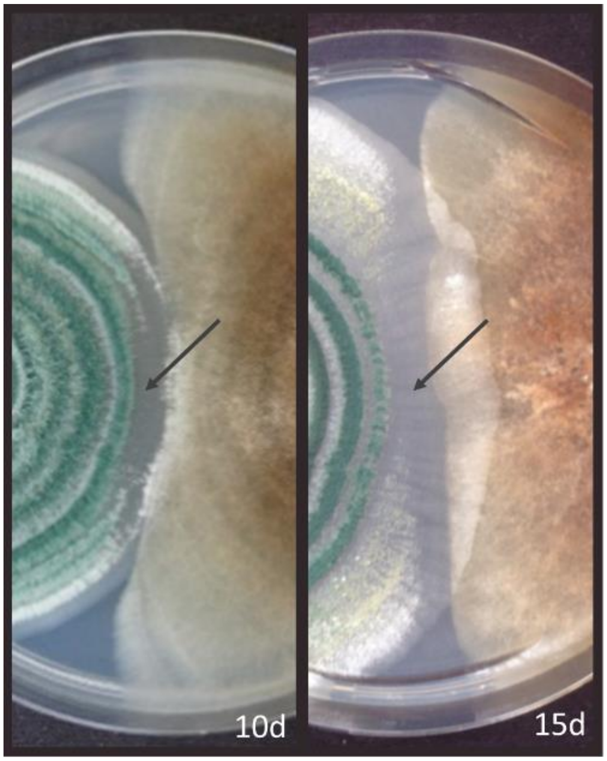

Supplementary figure 6: Zone of contact between the Δ*dfg5* deletion mutant and *Rhizoctonia solani* showing the reduced defense ability and conidiaton-free zone (arrows) in the Δ*dfg5* mutant upon host contact.

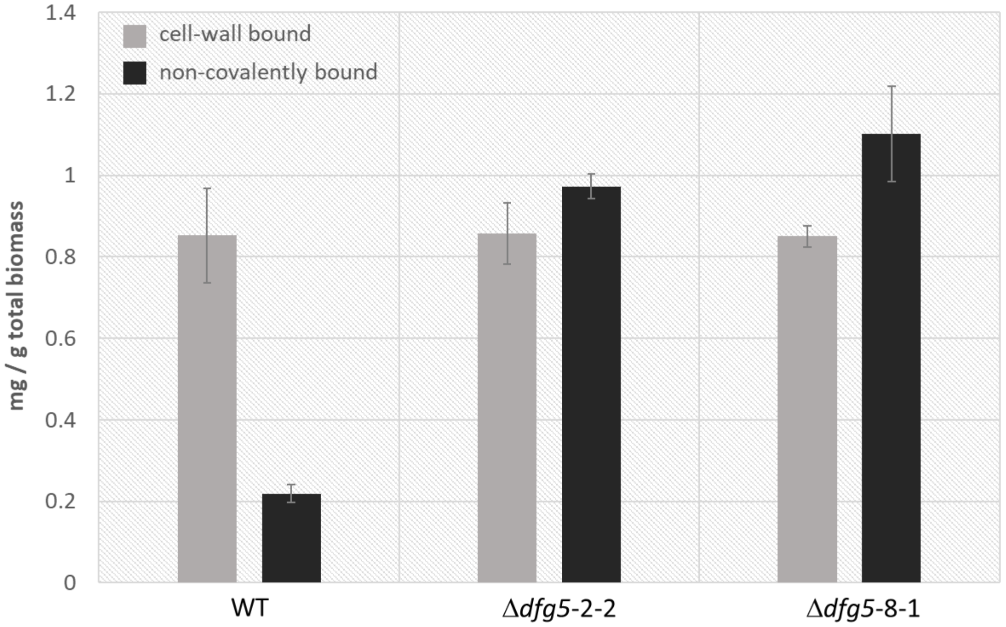

Supplementary figure 7: Total cell wall protein content and total release of non-covalently bound proteins from the washed cell walls of *dfg5* deletion mutants and wild type. The protein concentration was calculated using Bradford assay with BSA standard.

1.2 Legends to Supplementary Tables

Table S1: Oligonucleotides used for cloning, genotypic verification, sequencing and generation of transformation cassettes.

Table S2: Oligonucleotides used for the vector assembly using P*pki1*::*gpr1*-mEGFP::T*gpr1* construct, replacing the *gpr1*-mEGFP with *dfg5* gene downstream of the constitutively active *T. reesei pki1* promoter.

Table S3: RT-qPCR oligonucleotides used for differential gene expression analysis.

Table S4: Secreted protein hits differentially detected in *T. atroviride* wild type and Δ*dfg5*-8-1 analyzed by LC-ESI-MS and combining MASCOT search results of the five excised gel zones

Table S5: Identified proteins by LC-ESI-MS detected in *T. atroviride* wild type and Δ*dfg5*-8-1 in the individual gel zones.

1.3 Supplementary material references

Waterhouse, A., Bertoni, M., Bienert, S., Studer, G., Tauriello, G., Gumienny, R., Heer, F.T., de Beer, T.A.P., Rempfer, C., Bordoli, L., et al. (2018). SWISS-MODEL: homology modelling of protein structures and complexes. Nucleic Acids Res. *46*, W296–W303.
